# Wireless capsule endoscopy multiclass classification using three-dimensional deep convolutional neural network model

**DOI:** 10.1186/s12938-023-01186-9

**Published:** 2023-12-15

**Authors:** Mehrdokht Bordbar, Mohammad Sadegh Helfroush, Habibollah Danyali, Fardad Ejtehadi

**Affiliations:** 1 Department of Electrical Engineering, Shiraz University of Technology, Shiraz, Iran; 2 Department of Internal Medicine, Gastroenterohepatology Research Center, School of Medicine, Shiraz University of Medical Sciences, Shiraz, Iran

**Keywords:** Wireless capsule endoscopy, Image classification, Deep learning, 3D convolutional neural network

## Abstract

**Background:**

Wireless capsule endoscopy (WCE) is a patient-friendly and non-invasive technology that scans the whole of the gastrointestinal tract, including difficult-to-access regions like the small bowel. Major drawback of this technology is that the visual inspection of a large number of video frames produced during each examination makes the physician diagnosis process tedious and prone to error. Several computer-aided diagnosis (CAD) systems, such as deep network models, have been developed for the automatic recognition of abnormalities in WCE frames. Nevertheless, most of these studies have only focused on spatial information within individual WCE frames, missing the crucial temporal data within consecutive frames.

**Methods:**

In this article, an automatic multiclass classification system based on a three-dimensional deep convolutional neural network (3D-CNN) is proposed, which utilizes the spatiotemporal information to facilitate the WCE diagnosis process. The 3D-CNN model fed with a series of sequential WCE frames in contrast to the two-dimensional (2D) model, which exploits frames as independent ones. Moreover, the proposed 3D deep model is compared with some pre-trained networks. The proposed models are trained and evaluated with 29 subject WCE videos (14,691 frames before augmentation). The performance advantages of 3D-CNN over 2D-CNN and pre-trained networks are verified in terms of sensitivity, specificity, and accuracy.

**Results:**

3D-CNN outperforms the 2D technique in all evaluation metrics (sensitivity: 98.92 vs. 98.05, specificity: 99.50 vs. 86.94, accuracy: 99.20 vs. 92.60). In conclusion, a novel 3D-CNN model for lesion detection in WCE frames is proposed in this study.

**Conclusion:**

The results indicate the performance of 3D-CNN over 2D-CNN and some well-known pre-trained classifier networks. The proposed 3D-CNN model uses the rich temporal information in adjacent frames as well as spatial data to develop an accurate and efficient model.

**Supplementary Information:**

The online version contains supplementary material available at 10.1186/s12938-023-01186-9.

## Background

Wireless capsule endoscopy (WCE) is a non-invasive device that facilitates the examination of the gastrointestinal (GI) tract, especially those parts that cannot be screened by endoscopy and colonoscopy (small bowel). WCE transmits two images per second. After 8 h of passing through the digestive system, the data recorder saves approximately 57,000 frames. Physicians have to review all the frames, which takes up to 2 h [1]. However, a few of them include lesions. Therefore, an efficient computer-aided diagnosis (CAD) system for automatically recognizing abnormalities seems necessary to save time, improve detection accuracy, and facilitate the diagnosis.

CAD systems can divide into two groups: traditional methods and deep network models. The traditional CAD systems base their decisions on the features extracted from WCE images. For example, since bleeding occurs in most gastric diseases, color features have been recommended as a sensible choice in some research [2–4]. Some others have suggested textural features to detect polyps and tumors [5–8]. However, relevant feature extraction and appropriate feature selection take time and depend highly on the disease type, which is crucial in determining the output model [3]. The second step in traditional CAD systems is an automatic machine that makes a decision based on those features. Support vector machine (SVM) is one of the most suggested methods as a good classifier in literature [5, 6, 9, 10]. Some other studies have used different decision machines, such as Naive Bayes and K-mean algorithms [7].

Recently, deep learning models such as convolutional neural network (CNN) algorithms with hierarchy learning structures by building high-level features from low-level ones have been considered [11]. Deep neural networks try to figure out the solutions in terms of the problem concepts, in a manner that each concept builds on top of the others. This hierarchical learning scheme minimizes user intervention [12]. The main power of CNN lies in its deep architecture [13]. Using deep neural networks for detecting abnormalities in WCE frames is no exception [14].

Several attempts have been made to develop a reliable CAD system using deep network models to facilitate the WCE diagnostic procedures. For example, Tsuboi et al. have used the CNN system for the automatic detection of small bowel angioectasia in capsule endoscopy images [13]. Aoki et al., as another example, have used the same strategy as Tsuboi for erosions and ulcerations [15]. Rustam et al. have utilized a different architecture of deep neural networks for bleeding detection [16]. Jia et al. have compared deep neural network output with handcraft features for bleeding detection in wireless capsule endoscopy [14]. Similar work has done by Byren et al. for polyp detection [17]. Also, Caroppo et al. have classified WCE images into two categories (bleeding and non-bleeding) using three types of pre-trained CNN models [12]. Similarly, Kim et al. have used the Inception-Resnet-V2 model for the binary classification of frames containing a clinically significant lesion, such as inflamed mucosa, abnormal vascularity, or bleeding [18]. Additional file 1: Table S1 provides a comprehensive review of the progress made over the years in classifying WCE videos using deep neural networks [19–26].

Because the camera moves randomly in the GI tract, an anomaly may appear in some adjacent frames with different brightness, color scheme, texture, and structural form. Therefore, in practice, for sufficient assurance in diagnosis, gastroenterologists need to inspect a collection of nearby WCE frames collectively. This feature motivates us to propose a framework to imitate this diagnostic approach. We have developed a robust CAD system for anomaly detection on WCE videos by considering the fact that gastroenterologists do not base their diagnosis solely on one single WCE frame. We proposed that a suitable CNN classifier is required that not only keeps the potent two-dimensional (2D) features capability, but also incorporates the relationship between temporally adjacent frames. This concept is predominantly referred to as “video anomaly detection” in the literature.

The domain of “video anomaly detection” has witnessed substantial progress, particularly concerning surveillance videos [27]. Lately, the scope has broadened to encompass medical videos, such as endoscopy and colonoscopy, which pose their own distinct challenges [28]. The intricacy of anomaly detection in videos stems from the diverse appearances of anomalies and the potential for misleading complications. This complexity is further heightened when the background scene is dynamic. In “video anomaly detection”, identifying anomalies against a noisy and dynamic background can often lead to false positives, especially when the anomalies are small [29]. This situation is frequently encountered in endoscopic videos, introducing an additional layer of complexity to the problem compared to conventional surveillance systems. Furthermore, traditional object detection algorithms in images operate under the assumption that data are independent and identically distributed (i.i.d.). However, this assumption is not valid for videos where autocorrelation exists among sequential frames. To tackle these challenges (high false-positive rates in dynamic backgrounds with small anomalies, and dependency among images and features in time series), researchers have proposed spatiotemporal feature analysis [29–32]. This method aims to enhance the accuracy and reliability of anomaly detection in video data.

Even though 2D-CNNs succeed at capturing spatial features, they cannot capture the temporal information contained in three-dimensional (3D) data, such as videos. By applying convolution in three dimensions, 3D-CNNs can capture the temporal and spatial features present in the video data. The 3D convolution is attained by applying a 3D kernel to the cube created by stacking several consecutive frames together and extracting spatiotemporal information.

In video processing, a three-dimensional convolution technique was used for the first time to recognize human actions [33]. It worked effectively and performed better than other models that used recurrent neural networks to process data from the video's temporal dimension. The same approach can be applied to colonoscopy or endoscopy video analysis. Some literature has introduced a polyp-detecting algorithm in colonoscopy videos using a two-step process, including temporal information [34–36]. Their method initially extracts spatial features such as color, texture, and shape context to identify potential regions. Using independent 2D-CNNs, from each candidate region, the spatiotemporal pattern of the polyps is generated from three consecutive frames. Later studies investigated the performance of 3D networks over 2D ones for video datasets [37]. Polyps detection in colonoscopy videos using a 3D fully convolutional network (3DFCN) was first presented by Yu et al. [38]. In their study, a 3D-CNN was applied along with the colonoscopy video to extract the spatiotemporal features. The assessment findings demonstrated the superiority of 3D-CNN over 2D-CNN in learning illustrative spatiotemporal characteristics from colonoscopy videos. In addition, other techniques have been suggested for integrating spatial information with temporal dependencies between a series of video frames to offer useful information for detecting polyps [39, 40]. Boers et al. [41] have shown that the integration of spatiotemporal data from endoscopic videos improves and strengthens decision-making for the identification of esophageal cancer. They used Long Short-Term Memory (LSTM) and Gated Recurrent Unit (GRU) frameworks. Ghatwary et al. [42] have also demonstrated that 3D-CNN frameworks outperform 2D-CNN for learning spatiotemporal features for detecting esophageal abnormalities in endoscopic videos.

In addition, the efficacy of 3D-CNN for learning from spatiotemporal features in different types of videos has been confirmed. The 3D-CNN can successfully recognize human actions and moving objects in videos [33, 43]. Tran et al. [44] have demonstrated that employing 3D-CNNs to simulate the apparent motion in videos is more appropriate than learning spatial features simply by using 2D ConvNets. Moreover, Colleoni et al. [45] have proved the superiority of 3D-CNN frameworks over the 2D-CNN for detecting surgical-instrument and joint-connection from videos by using spatiotemporal features.

Furthermore, because the physician is the one who makes the final decision on a patient’s condition, even if a very accurate CAD system for abnormality detection exists, the physician must double-check those frames that the CAD system has classified as normal. However, the physician pays less attention to frames labeled as “normal” by the CAD system. Accordingly, a well-designed CAD system is the most sensitive one, and the reduction of specificity is not a crucial issue here. These features also motivate us to divide non-lesion frames into two distinct classes to increase the effectiveness of the proposed CAD system. To implement this proposed idea, a new class named “poor” is suggested for the multiclass classification approach.

Air bubbles, strong shadows, and other artifacts like mucus, bile, excrement, food, and dark fluids can obscure some WCE frames (to variable degrees). These frames contain patterns that differ from normal or lesion frames. This class includes those frames with not enough classification confidence to be labeled as normal. However, due to possible hidden lesions in the poor-quality images, the physician should scrutinize them. This class of frames is named “poor”. By doing this, we believe that the false negative rate is considerably reduced at the expense of reducing specificity. As a summary, our contribution in this article is to categorize WCE frames into three groups to improve overall sensitivity, whereas, to the best of our knowledge, prior CNN-based approaches have attempted to divide frames into two categories: “normal” and “abnormal” (abnormal class may include several cases such as ulcer, bleeding, polyp, cancer, and others).

To the best of our knowledge, no literature has been published that classifies WCE frames by learning spatiotemporal features from the 3D-CNN framework. In this paper, an efficient method to automatically classify different types of frames from WCE videos is presented. In a patient dataset, the performance of the proposed deep learning method is compared to that of the traditional 2D-CNN method.

## Results

The demographic and clinical data of the patients are given in Table 1. The dataset included the following lesions: ulcer, bleeding, ectasia, ampullae, aphthous ulcer, erosion, scar, telangiectasia, erythematous, xanthoma, white ulcer, and polyp. Samples of different types of lesions that were included in the dataset are presented in Additional file 1: Fig. S1.

**Table 1:** Demography and clinical data of patients undergoing capsule endoscopy

No. of cases	29
Age (years, mean and ranges)	(53.5, 14_94)
Gender	
Male (%)	66%
Type of disease	
1. Ulcer	7
2. Bleeding	3
3. Ectasia	3
4. Ampullae	2
5. Aphthous ulcer	2
6. Erosion	3
7. Scar	1
8. Telangiectasia	1
9. Erythematous	2
10. Xanthoma	2
11. White ulcer	1
12. Polyp	2

**Fig. 1:**
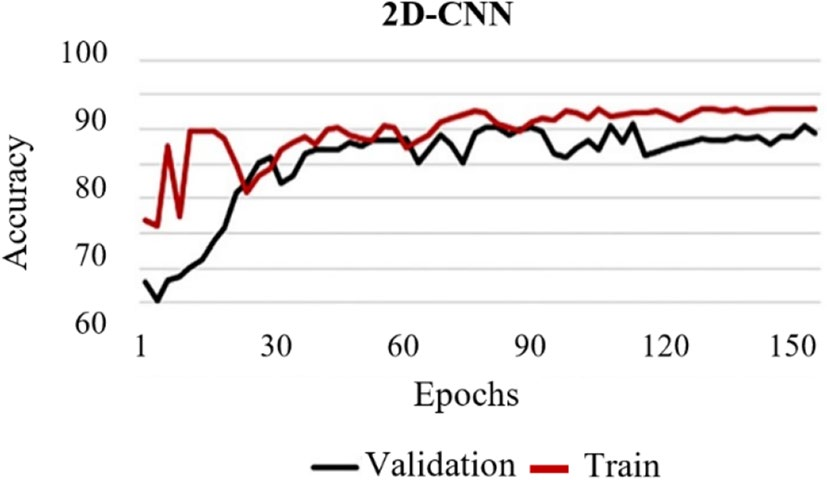
Accuracy of the proposed 2D-CNN model along different epochs

**Fig. 2:**
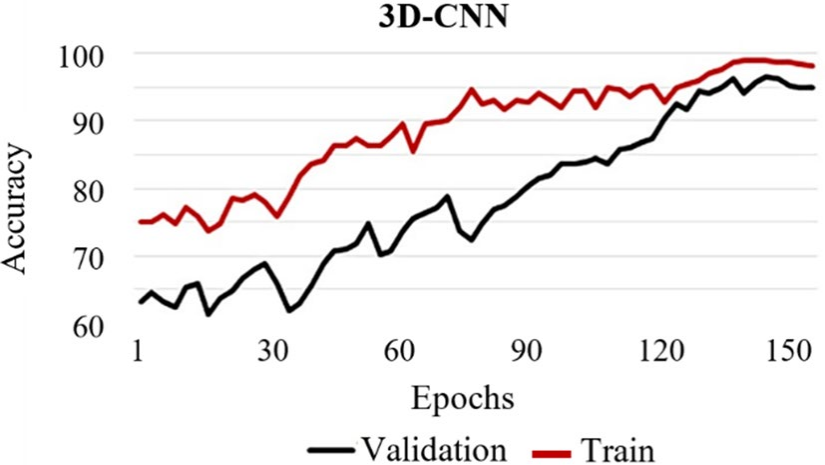
Accuracy of the proposed 3D-CNN model along different epochs

To select the appropriate number of epochs, the accuracy of the training and validation datasets for each epoch was saved and shown in Figs. 1 and 2 for the proposed 2D and 3D models separately. After 150 cycles, we found that the accuracy reached a plateau just before 150 epochs. In our study, the epoch number was chosen as 150, and the training took place for about 12 h for both 2D and 3D CNN models.

The results are provided on the test dataset for two evaluation scenarios: frame-based and disease-based approaches. Table 1 presents the confusion matrices for the proposed 2D and 3D CNN models, considering the frame-based scheme. The evaluation metrics extracted from Table 2 are listed in Table 3. All the evaluation metrics exhibit that the 3D deep neural network outperforms the 2D technique.

**Table 2:** Confusion matrices for the 2D and 3D proposed deep neural networks: frame-based scheme

		CNN Label		
		Lesion frames	Normal frames	Poor frames
		3D Net.–2D Net	3D Net.–2D Net	3D Net.–2D Net
Physician label	Lesion frames	586–570	26–44	8–6
	Normal frames	9–304	2567–2243	4–33
	Poor frames	2–15	3–8	2070—2052

**Table 3:** Evaluation metrics of binary classification for different deep neural network models: frame-based scheme

	3D	2D	AlexNet	Inception-V3	ResNet-18	SqueezeNet	DenseNet-201
Sensitivity (%)	98.92	98.05	79.03	77.74	77.58	81.12	85.48
Specificity (%)	99.50	86.94	98.62	98.22	98.74	98.70	99.22
PPV (%)	99.51	88.61	98.69	8546	89.23	89.34	93.63
NPV (%)	98.92	97.73	97.19	97.05	97.04	95.50	98.07
*F*_1_ score	99.21	92.95	97.93	90.89	92.97	92.32	95.80
Accuracy (%)	99.20	92.60	94.81	95.65	96.12	96.26	97.10

Nine patients with 11 various lesion sites within WCE videos were enrolled in the disease-based detection approach. The 2D network missed one of the 11 lesion sites, but the proposed 3D network discovered at least one frame from each disease site. The result reveals that the 3D-CNN has 100% sensitivity in the disease-based approach in this dataset.

Moreover, the proposed 3D and 2D CNN models were compared to five pre-trained models in classifying WCE images using our datasets. As shown in Table 3, pre-trained networks outperformed the 2D model, while the proposed 3D model had the highest accuracy. DenseNet had the best accuracy (97.10%) among the tested pre-trained networks, whereas AlexNet had the lowest (94.81%). The accuracy rankings of these pre-trained networks match those of previous studies on a different dataset [46].

Additionally, evaluation metrics for multiclass classification are presented in Table 4. According to the findings, the networks are more effective at recognizing poor frames than the other two classes. Furthermore, as expected, detecting lesion-containing frames is less sensitive than identifying normal frames.

**Table 4:** Evaluation metrics of multiclass classification for different deep neural network models: frame-based scheme

		3D	2D	AlexNet	Inception-V3	ResNet-18	SqueezeNet	DenseNet-201
Sensitivity (%)	Lesion	94.52	91.94	75.38	77.74	77.58	81.13	85.48
	Normal	99.50	86.94	97.72	96.89	97.74	97.78	99.10
	Poor	99.76	98.89	98.51	98.78	98.63	98.93	98.10
Specificity (%)	Lesion	99.76	93.15	98.62	98.23	98.75	98.70	99.22
	Normal	98.92	98.07	94.20	95.51	95.06	95.85	95.85
	Poor	99.62	98.78	99.14	98.71	98.90	99.22	99.84
PPV (%)	Lesion	98.16	64.12	88.61	85.46	89.24	89.34	93.64
	Normal	98.88	97.73	93.92	95.40	95.01	95.77	95.82
	Poor	99.42	98.13	98.70	98.02	98.30	98.78	99.75
NPV (%)	Lesion	99.27	98.86	96.57	97.05	97.05	97.50	98.07
	Normal	99.51	88.69	97.83	96.96	97.77	97.82	99.11
	Poor	99.84	99.28	99.02	99.21	99.12	99.31	98.79
*F*_1_ score	Lesion	98.71	77.79	92.42	90.89	92.98	93.24	95.81
	Normal	99.19	92.99	95.83	96.18	96.37	96.79	97.44
	Poor	99.63	98.70	98.86	98.61	98.71	99.05	99.27
Accuracy (%)	Lesion	99.15	93.00	95.73	95.81	96.24	96.62	97.60
	Normal	99.20	92.63	95.88	96.19	96.38	96.80	97.44
	Poor	99.68	98.82	98.89	98.74	98.80	99.10	99.16

The performance of the proposed 3D-CNN, as applied to the Kvasir-Capsule dataset [47], was evaluated and compared with the results of other studies. Table 5 presents these findings, juxtaposed with the results obtained from the same dataset in a study by Jain et al. [48]. In the referenced study, seven state-of-the-art deep networks were modeled and trained using the Kvasir-Capsule dataset, with their performance metrics also detailed in Table 5. Upon examination, it is evident that the proposed 3D-CNN network exhibits the highest performance metrics on average for the detection of various anomalies.

**Table 5:** Evaluating the proposed network in comparison with the state-of-the-art models tested by Jain et al. [48] for the detection of anomalies in the Kvasir-Capsule dataset

Model architecture	3D-CNN	2D-CNN	Simple 2D-CNN*	Weakly supervised CNN + iterative cluster unification*	VGG19 + InceptionV3 + ResNet50*	Dilated CNN*	Meta-feature parallel CNN*
Proposed by	This study	This study	Jia et al. [14]	Iakovidis et al. [49]	Caroppo et al. [12]	Goel et al. [50]	Hybrid CNN [48]
Sensitivity (%)	97	92	92	91	93	93	97
PPV (%)	98	89	87	89	96	93	97
*F*_1_ score	98	92	89	90	95	93	97
Accuracy (%)	98	93	90	91	95	93	97

* The evaluation metrics are reproduced from the original study of Jain et al. [48]

## Discussion

Fast and accurate GI disease detection can be attained using deep learning techniques on WCE videos as efficient building blocks. State-of-the-art architecture and models can be used to extend them. Deep learning techniques, however, should not be viewed as a comprehensive approach, because they also have some disadvantages when compared to more straightforward models. These include an increase in computational complexity and its dependence on the image quality of the training set, which results in a reduction of model generalizability. As an example, it has been demonstrated that when a well-trained deep neural network is exposed to unobserved external WCE data, its accuracy drops by around 10% [18].

Our results have demonstrated that the 3D deep neural network outperformed the 2D model in both the frame-based and disease-based schemes. The performance advantages of 3D-CNN could be attributed to various factors. First of all, 3D-CNN enables the inclusion of potentially relevant spatial (3D) context around the centering frame during training. During inference, based on various view angles and the brightness of a particular scene throughout succeeding frames, this model makes its own decisions. In contrast, 2D-CNN models are unable to use this additional information in the training phase, whereas during inference, they classify a single frame simply by taking the context of a WCE frame into account. Second, the slight differences in the model kernels and hyperparameters between 2 and 3D-CNN models may have contributed to the observed performance differences. It is crucial to understand how the time and space complexity trades off against the performance increase, which may or may not be clinically or statistically meaningful. In the models we applied, the 2D-CNN model, which is less complex, consist of approximately 8 million trainable parameters. This is significantly less compared to the 50 million parameters in the more complex 3D-CNN model. The reduced complexity of the 2D-CNN models offers several advantages. They are computationally more efficient, requiring fewer resources for training and prediction. They are also less likely to overfit the training data, leading to better generalization on unseen data. Furthermore, they are more data-efficient, requiring less data for effective training. However, the 3D-CNN may be advantageous in detecting GI diseases using WCE videos based on the findings. Additionally, in this study, a multiclass approach has been utilized to maximize the classification strengths of both 2D and 3D-CNN. Since classification algorithms frequently fail to correctly classify WCE frames that are masked by strong shadows, air bubbles, and other artifacts, we have considered creating a separate class for them.

**Fig. 3:**
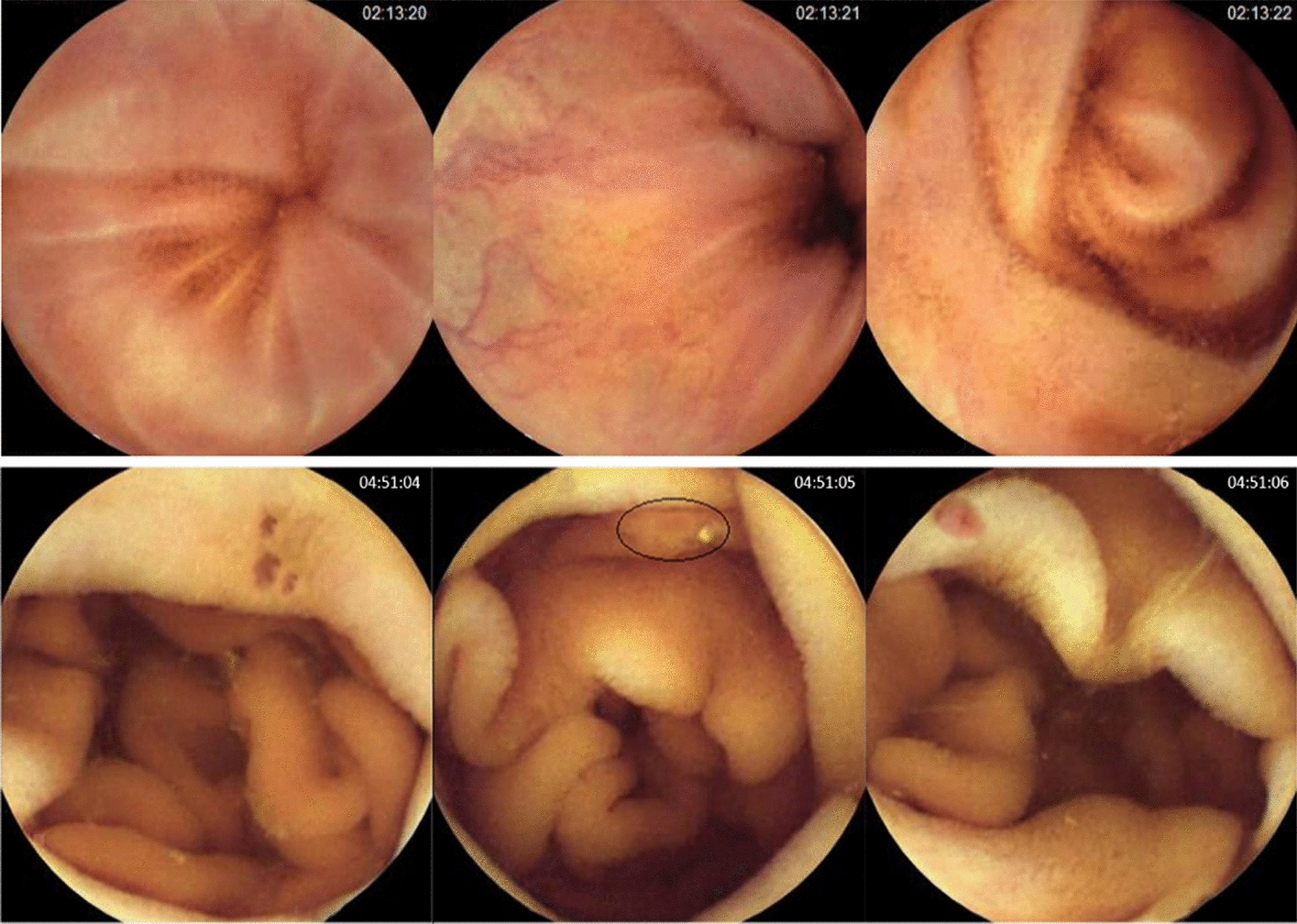
Two examples of frames (second column) that the 2D-CNN misclassified when the 3D-CNN correctly classified them. First row: normal frame incorrectly classified as lesion frame. Second row: lesion frame incorrectly classified as normal

Figure 3 shows a few instances of frames where the proposed 2D model misclassified them while the proposed 3D model correctly classified them. As is shown in the center of the first row in Fig. 3, on the left side of the image, a pattern similar to an ulcer incorrectly has appeared because the camera lighting has enhanced the color of the superficial vessels there. However, this pattern has vanished in the adjacent frames. While the 2D-CNN was unable to recognize that this pattern is unrelated to ulcer lesions, the 3D-CNN did.

In Fig. 3, there is an erosion lesion instance in the WCE frame in the center frame of the second row. However, the lesion has been concealed by the wrinkle patterns of the GI tract. Although the CNN system might be able to detect this lesion in the adjacent frames, it is challenging to detect it in this particular frame. In this sample, the 3D-CNN detected the presence of a lesion in this GI site, but the 2D-CNN did not.

Other findings show that the pre-trained networks outperform a 2D simple CNN model for WCE image classification. This benefit could be associated with either their structure or pre-trained weights. To discover the source of their advantages, we also trained those five models from scratch (the results of this part are not presented due to space constraints). We found that the classification accuracy would be low if those structures were trained from scratch with our dataset. Also, it was anticipated that Inception-V3 would outperform ResNet-18 and SqueezeNet; nevertheless, because those two latter architectures are made to be effective with fewer parameters, they show higher performance with the sparse data in this study. The conclusion is that maybe larger datasets were required to accurately train too deep network structures to converge. Consequently, we reasoned that rather than their deep architectures, their advantages over ordinary 2D-CNN on our dataset came from their pre-trained weights.

Several publicly available datasets exist, such as “Kvasir-Capsule” [47], “KID Project” [22], “EndoSLAM” [51], and “Bleeding Dataset” [52]. Those datasets that include videos, which are beneficial for this project, are limited to approximately 40 cases. Consequently, at the start of the study, we chose to build and train our models using our in-house dataset, which consists of WCE videos from 29 patients. For a thorough comparison with state-of-the-art deep network models, we trained and evaluated our proposed models using the Kvasir-Capsule dataset. The results, presented in Table 5, demonstrate the superiority of the 3D-CNN model in utilizing spatiotemporal features. This approach outperforms previously reported state-of-the-art deep network models in classifying frames from Kvasir-Capsule videos. Howbeit, we recommend that future research could consider testing the 3D-CNN models with others publicly available WCE video datasets.

## Limitations

There are some limitations of this study. First, the ground truth for this study was established by one physician reviewing the WCE videos. Considering how challenging it is for a gastroenterologist to interpret a large number of WCE images for lesion detection, there may be some errors with the interpretation results; however, the errors and their impact on the results should be very modest, and they should not change our conclusion. Second, the three-class strategy that was developed might not be the best approach to the WCE frames classification issue. As we previously stated, we focus on clarifying the performance differences between 3D-CNN and 2D-CNN in classification, not on creating classification models. Therefore, we did not perform an in-depth comparison with other CNN architectures that were readily available. However, our developed 2D-CNN models showed comparable results to other methods [16, 46, 53], and the 3D-CNN exhibits a promising classification performance. Third, it is possible that the CNN model's hyperparameters are not sufficiently tuned for this specific classification purpose. Several variables, including batch size, optimizer selection, learning rate, and kernel numbers, are used in the training of a CNN model. The model's performance may be altered by changing these settings. Fourth, since there were fewer lesion frames compared to the other two groups, the dataset was imbalanced. To counteract this issue, we used a weighted objective function. However, there is an additional imbalanced dataset dealing with solutions that may be explored, including employing a large multicenter dataset including different lesion types.

The fifth limitation relates to optimizing hyperparameters and evaluating network performance. The network training was performed on over 146,000 instances after the augmentation phase. We also had to tune the hyperparameters of the two proposed CNN models (2D-CNN and 3D-CNN). To comprehensively estimate the performance of proposed methods and improve model tuning, robust approaches such as bootstrap and cross-validation techniques could be utilized. However, these techniques are highly computationally intensive, and due to limitations in system memory and processing power (using Google Colab GPU), we were unable to use it. Nonetheless, it has been demonstrated that on colossal datasets, cross-validation techniques may not significantly improve classification evaluation. For instance, using k-fold cross-validation on different colossal datasets has improved the accuracy of classification systems by less than 1% in various literature [54].

However, cross-validation can help reduce the bias and variance of estimated performance compared to simply splitting the dataset into training, validation and testing sets. When splitting the dataset into training, and testing sets, there is a risk of overfitting and underestimating the performance on the testing set, which can lead to high variance in the estimated performance. However, in the above examples, it has been shown that on colossal datasets, the underestimation is less than 1%. On the other hand, if the split is not representative of the underlying data distribution, the estimated performance may be biased. To reduce this drawback, we split the datasets based on the patients’ WCE videos instead of frames. In this way, the testing dataset is somehow individual from the training and validation sets, and can lessen the computed performance's bias toward the optimal system performance.

Besides the limitations, there are some other suggestions for further work. To the best of our knowledge, no existing literature classifies WCE frames using the 3D-CNN framework to learn spatiotemporal features. As this is the first application of 3D-CNN in this field, we chose the basic model of 3D-CNN to highlight the advantage of spatiotemporal analysis over spatial features. However, there are innovative 3D deep network architectures such as R(2 + 1)D [55], SlowFast [56], 2D-3D CNN [57], late temporal 3D-CNN [58], that might outperform the basic 3D-CNN model in certain aspects. Despite this, enhancing the 3D-CNN model architecture could potentially skew the system’s performance from our proposed hypothesis (the benefits of spatiotemporal features) toward the novelty of the architecture itself. Therefore, we recommend assessing the performance of various novel 3D-CNN models compared to the basic architecture in future work.

## Conclusions

We developed a CNN framework to perform the multiclass classification of frames (lesion frame, normal, and poor images) depicted on WCE videos. The novel 3D-CNN proposed in this article classifies a frame by using a series of consecutive frames from WCE videos. This concept develops a more efficient CNN model that uses temporal in addition to spatial information, as opposed to 2D-CNN models that only use spatial information. We implemented the 2D and 3D versions of this framework to clarify whether and to what extent a 3D-CNN outperforms its 2D counterpart. Our results on a relatively large and diverse dataset showed that the proposed 2D-CNN and 3D-CNN models gain high sensitivity to detect those frames' content GI lesion, albeit the latter one yielded much better performance (7% improvement in accuracy). We believe the existing 2D-CNN architectures may not fully utilize the rich information in succeeding frames. Additional investigative efforts may be needed to develop sophisticated CNN models to maximize the rich information in adjacent frames instead of exploiting them as independent frames.

## Materials and methods

### Datasets

The dataset was collected retrospectively from the gastroenterology ward at Namazi Hospital, Shiraz, Iran. It contains WCE videos from 29 individuals. All metadata have been eliminated and the files have been renamed using a randomly generated code. Consequently, the dataset is fully anonymized, as sanctioned by the Privacy Data Protection Authority and in compliance with the pertinent guidelines and regulations of the Regional Research Committee of the Shiraz University of Medical Sciences. The Regional Research Committee of the Shiraz University of Medical Sciences has granted approval to analyze anonymous images for this project without requiring consent from participants. This project was exempted from further approval by the Iran National Committee for Ethics in Biomedical Research.

**Fig. 4:**
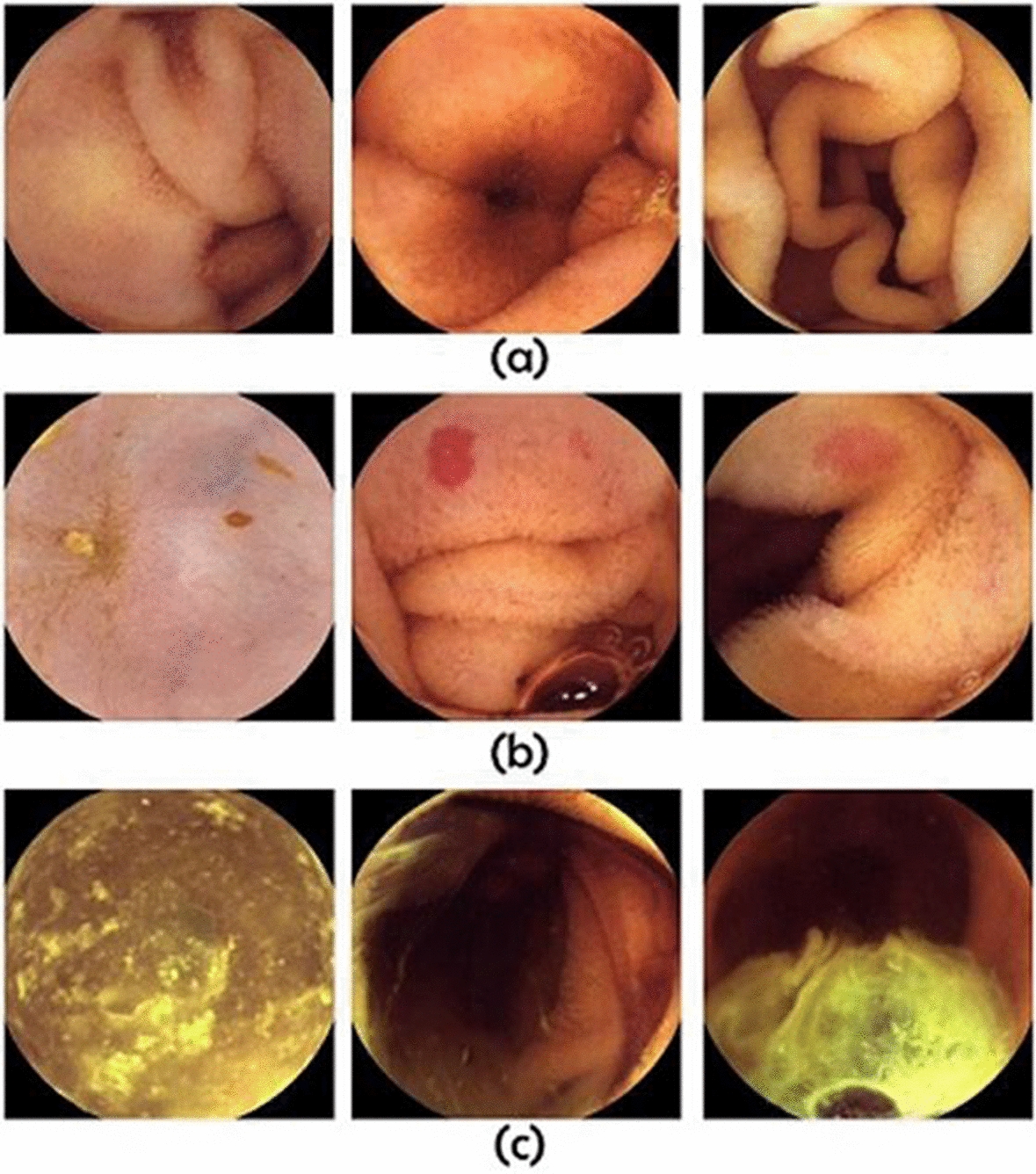
Representations of some sample data. **a** Normal frames. **b** Abnormal frames. **c** Poor frames

Videos have been captured with the PillCam™ SB3 (Medtronic Japan) capsule at a rate of two frames per second. We have extracted 14,691 frames (7779 as normal frames, 1906 as lesion frames, and 5006 as poor frames) in total. Figure 4 shows some dataset sample frames (more samples of different types of lesion frames are shown in Additional file 1: Fig. S1). An expert physician has examined and labeled all of the data.

**Fig. 5:**
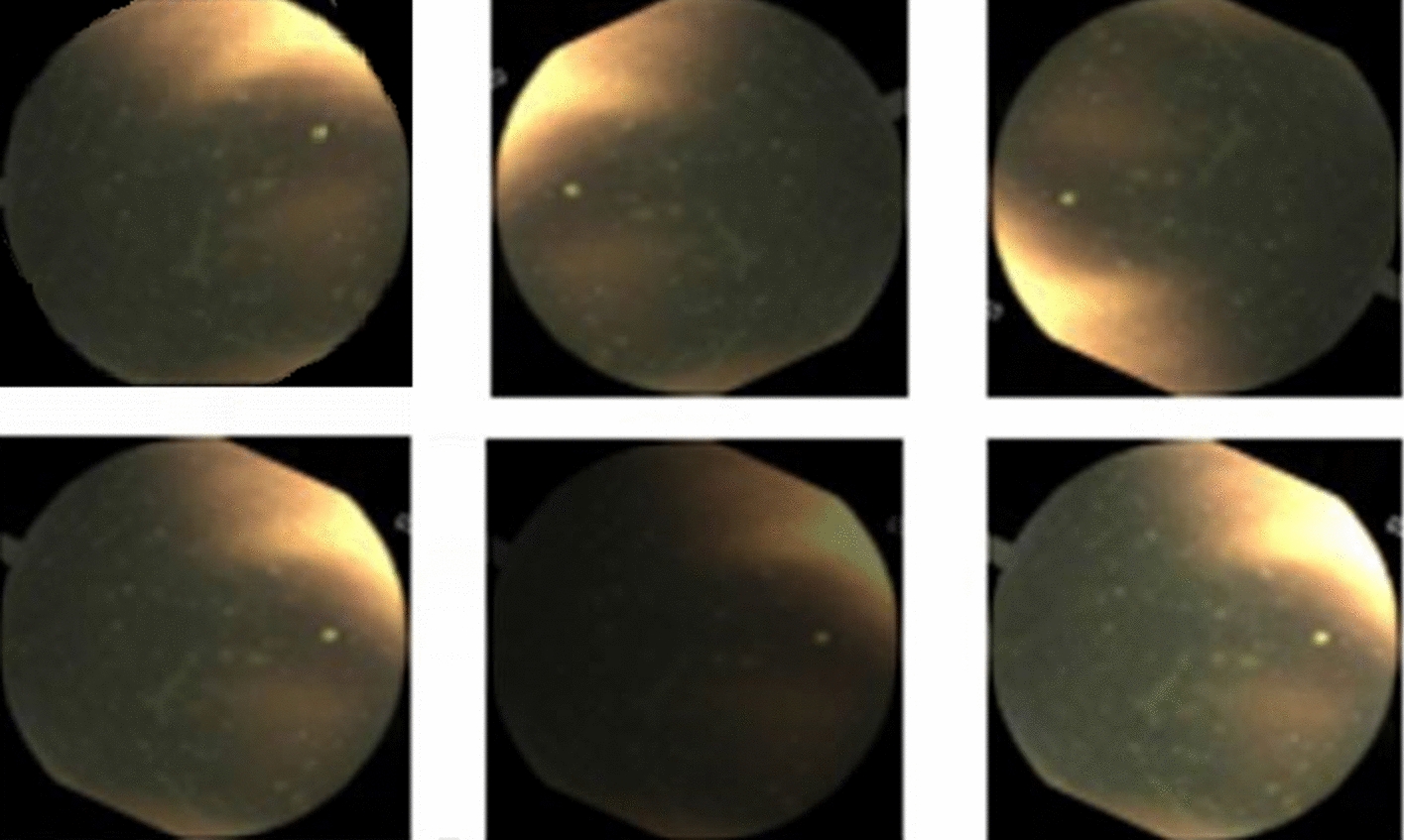
An example of data augmentation

The dataset underwent four steps of preprocessing steps prior to being input into the convolutional neural network (CNN). Initially, intensity normalization was performed on the entire dataset by rescaling the intensity values by their standard deviation, ensuring that the target values have a mean of 0 and a standard deviation of 1. Subsequently, all images were cropped to their smallest square form to confine the image matrix to the active circular region, thereby minimizing the surrounding black background. The intensity values of this black background, which contains no information, were maintained at zero following the rescaling phase. This ensured that their kernel values remained inactive during network implementation. However, the reduction of the black background contributed to decreased memory and CPU usage. Furthermore, all images resized to a dimension of 224 × 224 to optimize memory usage. The resulting image dimension was 224 × 224 × 3, with the third dimension representing the RGB color space. The cubic B-spline method was utilized for image resizing. Finally, during the data augmentation phase, it was imperative to utilize techniques that guaranteed the generated images were representative of those that could be obtained from wireless capsule endoscopy (WCE) systems. This involved the application of rotation (limited to 15 degrees), horizontal and vertical flipping, as well as brightness adjustments (limited to 20%) in this study. Figure 5 shows an example of data augmentation. Twenty patients (75%) were randomly selected for training, and nine remaining patients (25%) were considered as the test dataset. In the training dataset, 25 percent of frames (randomly selected) were used for network validation.

In order to facilitate a comprehensive comparison of the performance of the 3D-CNN model proposed in this study with the findings reported in other scholarly articles, we have conducted an analysis of our two basic networks (2D-CNN and 3D-CNN) using images derived from the Kvasir-Capsule publicity available dataset [47] . The dataset under discussion comprises 43 WCE videos. In total, these videos include 3491 lesion frames (spanning 42 unique lesion timepoints), 34,338 normal frames, and 2906 poor frames (categorized as “Reduced Mucosal View” within the dataset). The lesion frames contain eight distinct classes of anomalies, each labeled accordingly: ‘Angiectasia’ (appearing 9 times), ‘Blood–fresh’ (appearing 3 times), ‘Blood–hematin’ (appearing once), ‘Erosion’ (appearing 11 times), ‘Erythema’ (appearing 5 times), ‘Lymphangiectasia’ (appearing 4 times), ‘Polyp’ (appearing once), and ‘Ulcer’ (appearing 5 times). The preprocessing phase includes normalization and resizing of the images. Consistent with the previous section, the final image is resized to dimensions of 224 × 224 × 3, where the third dimension represents the RGB color space. For the 3D-CNN models, 16 consecutive frames are inputted together. The networks’ weights trained with the in-house dataset are mapped onto the network used in this section. During the fine-tuning of the Kvasir dataset, all weights, except those belonging to the dense layers, were frozen. For the training of the networks, frames from 30 randomly selected videos were used, while frames from the remaining 13 videos were used for validation.

### 3D-CNN versus 2D-CNN framework

The input layer of a 2D convolution operator can have multiple channels, such as RGB color images or intermediate layers in a deep network. In this case, multiple kernels can be applied to each channel separately. The value after such a convolution layer can be written as [59]:

1 $${V}_{{C}_{i}}\left(x,y\right)={\text{max}}\left(0,{\sum }_{i=1}^{N}{K}_{{c}_{i}}\left(x,y\right)*{I}_{{C}_{i}}\left(x,y\right) \right),$$

where max(.) is an elementwise rectified linear unit (ReLU) activation function, and (*) represents the convolution operator. ${c}_{i}$ represents the *i*^th^ channel number, and (*x*, *y*) represents the pixel position. *K* is a 3D array kernel that distributes weights between an input value in channel *i*^th^ to its corresponding channel in (*x*, *y*) position.

**Fig. 6:**
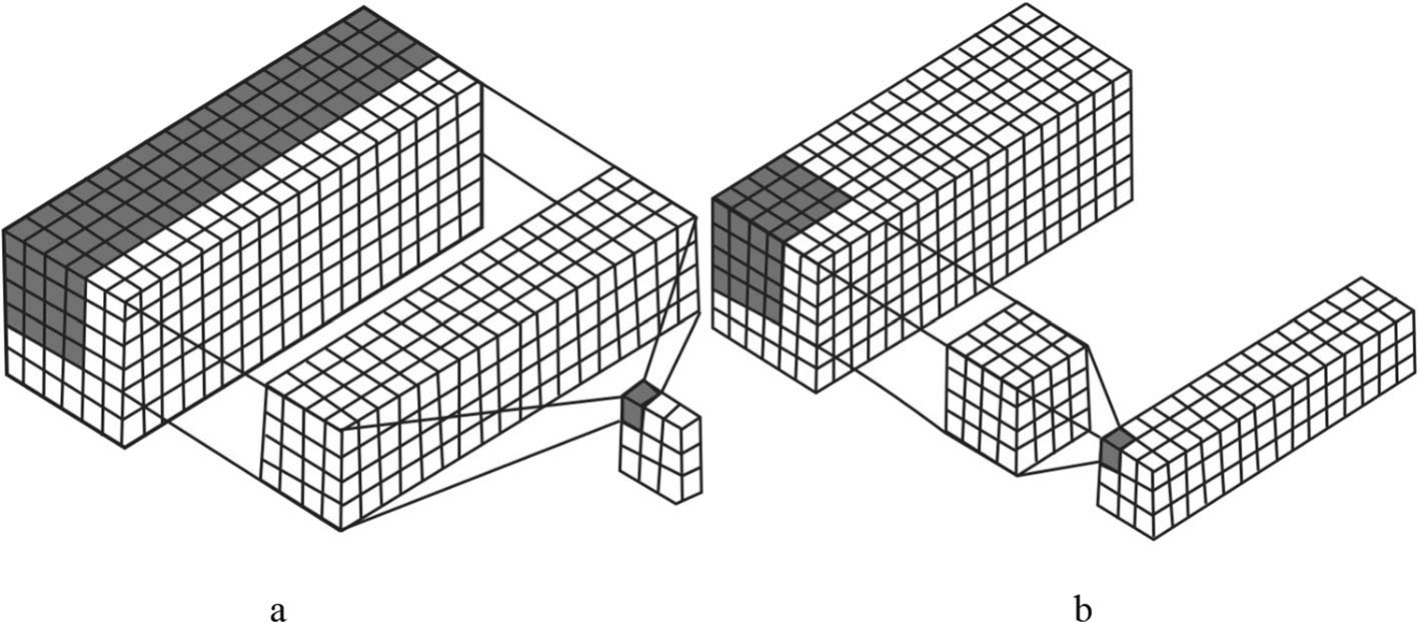
The difference between 2 and 3D convolution. **a** How 2D convolution performs on 3D data. **b** How 3D convolution applies cubic kernel to the 3D data

As Eq. (1) describes, the output of a 2D-convolutional layer for multi-channel input data will be the sum of all input channels multiplied by corresponding kernel weights. Therefore, a 2D convolution operation cannot extract any hidden relationships between different channels since it moves only in two directions represented by (x, y). It causes the neural network to lose information in the depth direction, whereas in video data, this relates to temporal information (see Fig. 6a). For the 2D color input images, it is not a problem since the neural network works with 2D data. However, when the issue contains video input data, it is better to consider it as 3D data, and thus, it should be described and approximated in three directions. To make this possible, 3D convolution operation can be used.

In contrast to the 2D convolution, the depth of the kernel size in the 3D convolutional operation is smaller than the input data depth. Therefore, the sliding window kernel extracts entangled information from all three directions as a cube (see Fig. 6b). Formally, 3D convolution in a position x, y, t at the i^th^ channel can rewrite from Eq. (1) as [35].

2 $${V}_{{C}_{i}}\left(x,y,t\right)={\text{max}}\left(0,{\sum }_{i=1}^{N}{K}_{{c}_{i}}\left(x,y,t\right)*{I}_{{C}_{i}}\left(x,y,t\right)\right),$$

where *x*, *y*, and *t* are the coordinates of the three-dimensional kernel of convolution operation; *K* is the kernel; I is 3D data on the *i*^th^ channel. The behavior of a 3D convolution looks like a 2D convolution operation, and for proper architectural design, the same concepts for the input data should be considered, such as shared weights and local receptive fields. In summary, the only difference is that the 3D convolution operation is applied to the cube and uses a smaller cube as a kernel; meanwhile, the 2D convolution operation just sums up all the values in the third dimension. The visual differences between them are shown in Fig. 6.

### 2D-CNN network architecture

The proposed CNN was developed in Python v3.7 environment and Pytorch 1.12.1 + cu113 using Google Colab GPU (Tesla K80 12 GB GDDR5 VRAM). After exploring various architectures, a CNN network model with five convolutional layers was heuristically proposed. Since the lesion structures may be differentiated in a small size within the 224 × 224 image size, a 3 × 3 convolutional kernel with one-pixel padding was utilized throughout all layers. Features were extracted using 32 kernels in the first layer, while the remaining levels used 64, 128, 256, and 512 kernels, respectively.

We empirically found that the skipped connections between layers did not increase overall system performance. It can be explained by the two following reasons. First, the low-level features of the three classes of images (normal, lesion, and poor frames) are nearly identical, while lesions are more distinguishable with high abstract features. Second, in this study, we are only searching for the appearance of a lesion in the frames, not its location.

**Fig. 7:**
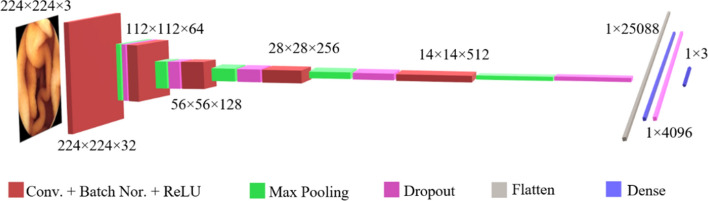
The architecture of the employed 2D-CNN

Batch normalization was connected to each convolutional layer, and a ReLU was used as an activation function. The ReLU function performs nonlinear decision-making with a good performance and a fast-learning rate. A max-pooling layer with 2 × 2 kernel size and two pixels striding was located after each CNN layer. Pooling layers make the network robust to spatial invariance while significantly lowering the computation cost. The Softmax layer was placed at the final layer to act as the multinomial logistic regression decision-making machine. Softmax is always placed after the dense layer since it reflects a probability distribution from a large number of features. Softmax itself is a dense layer with the same number of output nodes as classes. This layer maps the given neural network features $n\left(x\right):$ extracted from input $x$ to the softmax output probability value $\sigma \left(n\left(x\right)\right):{R}^{N}\to {R}^{M}$, where *N* is the number of extracted features at the last fully connected layer, and *M* is the number of classes, which is three in this project. It is defined as:

3 $$\sigma {\left(n(x)\right)}_{y}=\frac{{{\exp}}\left(n{(x)}_{y}\right)}{{\sum }_{{y}^{\prime}}^{M}{{\exp}}\left(n{(x)}_{{y}^{\prime}}\right)},$$

where *y* represents the class label. Figure 7 presents the architecture of the employed 2D-CNN in the current study.

### 2D-CNN network implementation details

The 2D-CNN was trained with 146,628 frames (after augmentation) of WCE with 224 × 224 × 3 image size. Batch size 32 was used, and the following implementation settings were selected: learning rate = 0.0001, sample per volume = 1, optimizer = Adam, loss function = cross-entropy, and decay rate = 0.0001. Dropout rate of 0.4 has been employed to make the model more robust. The dropout also gets the network rid of simple dependencies between the neurons. Since the number of lesion frames was much smaller than for the other two groups, an appropriate weighting factor for lesion frames was used in the cost function to deal with the imbalanced dataset. Accordingly, the weighted cross-entropy loss function for a Softmax output can be written as below for imbalanced input data:

4 $$L=-{\sum }_{i=1}^{M}{w}_{i}\times {y}_{i}\times {\text{log}}\left(\frac{{\text{exp}}\left({n\left(x\right)y}_{i}\right)}{{\sum }_{{y}^{\prime}}^{M}{\text{exp}}\left({n\left(x\right)}_{{y}^{\prime}}\right)}\right),$$

where ${w}_{i}$ and ${y}_{i}$ represent the arbitrary weight and class label for *i*th class, respectively. To minimize the loss function in the backpropagation phase with the gradient descent method, the gradient of the loss function was expressed as below:

5 $$\frac{\partial (L)}{\partial (X)}={\sum }_{i=1}^{M}\sigma {(n(x))}_{{y}_{i}}.$$

Within Additional file 1: Table S2 offers a summary of the proposed characteristics of the proposed 2D-CNN.

### 3D-CNN network architecture and implementation details

**Fig. 8:**
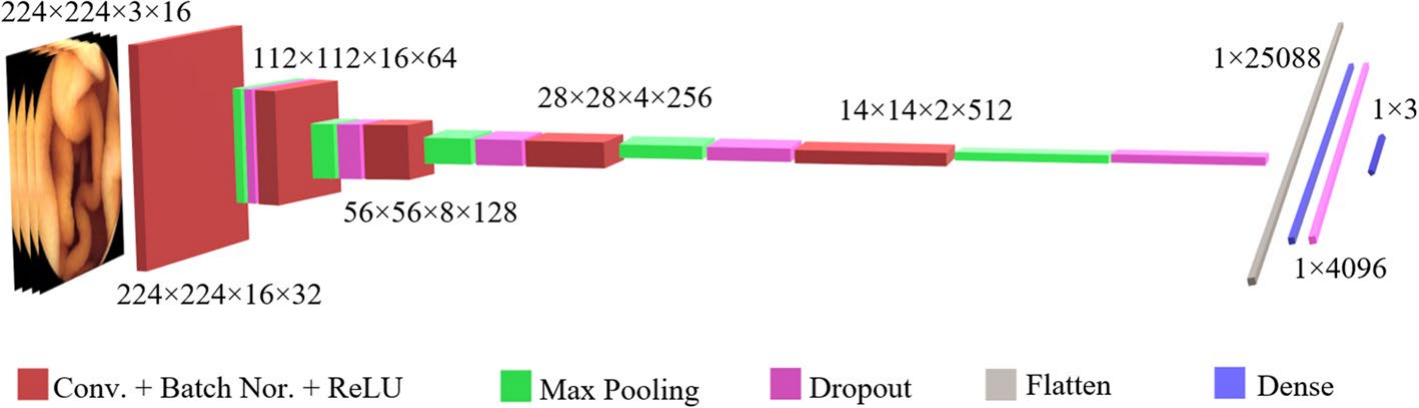
The architecture of the employed 3D-CNN

The 3D-CNN network had the same layer depth as the 2D-CNN network. Although, the input layer consisted of 16 adjacent color images. The 3D-CNN had the same number of kernels in each layer, batch normalization, activation function, and striding as the 2D-CNN. The convolutional kernel size was 3 × 3 × 3 voxels in the 3D-CNN. After the first convolutional layer, a max-pooling layer with a kernel size of 2 × 2 × 1 protected the temporal information at low-level features. Four later convolutional layers were followed by a 2 × 2 × 2 pooling layer. Every other detail of the 3D-CNN implementation was identical to the 2D-CNN. Figure 8 presents the architecture of the employed 3D-CNN in the current study. Additional file 1: Table S3 provides an overview of the characteristics of the proposed 3D-CNN.

### Pre-trained networks

To better illustrate the superior performance of our proposed 3D-CNN network (which utilizes spatiotemporal features) over 2D networks (that solely use spatial features), we have compared it with several of the most commonly used deep neural network classifiers. The integration of 3D structures, which utilize spatiotemporal features, can be incorporated into numerous state-of-the-art models to enhance their performance. Given the impracticality of testing the hypothesis on various novel models, we chose to demonstrate the benefits of the basic CNN spatiotemporal system using the most conventional deep network classifier. These networks have been previously employed in the classification of wireless capsule endoscopy (WCE) [12, 19, 24, 46, 60, 61] and various other benchmark image classification datasets [62–65]. This comparative study aims to provide a comprehensive understanding of the effectiveness of incorporating spatiotemporal features in image classification tasks.

We have implemented five different types of pre-trained networks called AlexNet, Inception-V3, ResNet-18, SqueezeNet, and DensNet. While each architecture has its unique specifications, they share many commonalities in their approach to image classification, including the use of CNNs, deep layer stacking, preprocessing, ReLU activation functions, and regularization techniques. Additionally, the different architectures often build upon and improve upon the methods used in previous architectures. Transfer learning was used to fine-tune the pre-trained deep neural networks on the training dataset. During the fine-tuning, all weights except those belonging to the dense layers were frozen. These networks were also trained with 150 epochs. In the following, we will briefly explain the characteristics of these networks and articulate the unique specification of each.

### AlexNet

The first architecture designed for image classification that used successive convolutional layers was AlexNet. It contains eight layers (5 convolutional layers and three fully connected layers) [66]. It won the ImageNet Large Scale Visual Recognition Challenge (ILSVRC) 2012. AlexNet achieved a top-5 error rate of 15.3% on the ImageNet dataset. Except for the last layer, where a Softmax with a distribution over the three class labels was applied, ReLU activation was performed at the end of each layer. In the first two completely linked layers, dropout was employed. Max-pooling was used after the first, second, and fifth convolutional layers. The neurons in the fully connected layers were connected to all neurons in the previous layer.

### Inception-V3

The data may overfit when numerous deep convolutional layers are used in a model. The inception model makes use of the concept of having multiple kernels of various sizes on the same level to prevent this [67]. Therefore, in the inception models, there are parallel layers rather than deep layers, which makes the model broader rather than deeper. Inception-V3 achieved a top-5 error rate of 3.5% on the ImageNet dataset. In this work, Inception-V3 contained 22 layers (nine convolution layers, four max-pooling layers, and nine linearly stacked inception modules). At the end of the previous inception module, the average pooling was implemented. Since 3 × 3 and 5 × 5 kernel sizes create complexity and expense, the 1 × 1 kernel was employed at the first layer. The number of parameters in the Inception-V3 model was double that of the AlexNet model.

### ResNet-18

Convolutional neural networks are the foundation of the ResNet-18 model, enabling a smooth gradient flow. The ResNet-18 model skips one or more layers when using the identity shortcut link. Enabling a skipped connection to the network's initial layers will make gradient updates considerably more straightforward for those layers [68]. It achieved state-of-the-art performance on the ImageNet dataset when it was introduced. ResNet-18 achieved a top-5 error rate of 5.6% on the ImageNet dataset. One fully connected layer and 17 convolutional layers make up the ResNet model.

### SqueezeNet

SqueezeNet provides a comparable result using more compact CNN architectures [69]. SqueezeNet is designed to be lightweight and efficient, with fewer parameters than other architectures. It achieved a top-5 error rate of 4.8% on the ImageNet dataset, with only 4.8 million parameters. It comprises a single convolution layer first (called conv1), then eight fire modules (called fire8), and a final convolution layer (called conv10). A squeeze convolution layer, which only contains a 1 × 1 kernel, feeds into an expand layer within a fire module. The expand layer includes a combination of 1 × 1 and 3 × 3 convolution kernels. From the beginning to the end of the model, the number of kernels per fire module is steadily raised. Max-pooling with a stride of 2 is typically applied after layers conv1, fire4, fire8, and conv10. More comprehensive information about the specific architecture of this network and the unique characteristics of its modules can be found in [69].

### DenseNet-201

DenseNet is a considerably deeper network with several appealing benefits: it overcomes the vanishing-gradient problem, improves feature dispersion, promotes feature reuse, and significantly reduces the number of parameters [70]. The name densely connected convolutional network refers to its architecture, in which each layer is directly connected to every other layer. The skipped connection in DenseNet does not sum the layer's output feature maps with the incoming feature maps as ResNet does, but instead concatenates them. DenseNet-201 achieved a top-5 error rate of 3.6% on the ImageNet dataset. DensNet-201 consists of four dense blocks with 6, 12, 48, and 32 convolution layers, respectively. Other hyperparameters, which have utilized in this paper, have been set in accordance with reference [71].

In summary, among these well-known classifier deep network models, AlexNet was a pioneering architecture that introduced several innovations, but has been surpassed by more recent architectures performance. Inception-V3 and DenseNet-201 have achieved state-of-the-art performance on image classification benchmarks with the lowest top-5 error rates on the ImageNet dataset. ResNet-18 and SqueezeNet have fewer parameters than other architectures and are intended to be lightweight and reliable.

### Evaluation metrics

The performance of the proposed deep neural network classifiers was measured on the test dataset using a confusion matrix. From confusion matrices, sensitivity, specificity, positive predictive value (PPV), negative predictive value (NPV), *F*_1_ score, and total accuracy were computed. These parameters are expressed as shown below:

6 $${\text{Sensitivity}}=\frac{{\text{TP}}}{{\text{TP}}+{\text{FN}}},$$

7 $${\text{Specificity}}=\frac{{\text{TN}}}{{\text{TN}}+{\text{FP}}},$$

8 $${\text{PPV}}=\frac{{\text{TP}}}{{\text{TP}}+{\text{FP}}},$$

9 $${\text{NPV}}=\frac{{\text{TN}}}{{\text{TN}}+{\text{FN}}},$$

10 $${F}_{1}{\text{score}}=2 \times \frac{{\text{PPV}} \cdot {\text{NPV}}}{{\text{PPV}}+{\text{NPV}}},$$

11 $${\text{Accuracy}}=\frac{{\text{TP}}+{\text{TN}}}{{\text{TP}}+{\text{TN}}+{\text{FP}}+{\text{FN}}},$$

where TP is the number of cases in which both the physician's label and model's prediction are as the lesion or poor; TN is the number of cases in which the model correctly assigns the label of “normal”; FP is the number of cases in which the model predicts a lesion or poor, but the physician's label is normal; FN is the number of cases in which the model predicts as normal, but the physician's label is lesion or poor.

The proposed idea was assessed with two evaluation scenarios: frame-based and disease-based approaches. In the frame-based approach, the strength of models in classifying each frame as independent inputs were evaluated. The mentioned evaluation metrics were computed for this approach.

However, the main objective of a CAD system is to locate the site of any potential disease within a patient's WCE video. Any disease would usually manifest itself by some lesions in more than ten consecutive frames. The diseased region within the WCE video is identified if the CAD system finds at least one frame within the adjacent lesion frames. Consequently, we defined a disease-based strategy to see whether the competing models can detect the site of the lesions that appear within the WCE video. To do so, the physician labeled 11 distinct disease appearance time points among nine patients in the test dataset. If the CNN model recognizes even one frame that belongs to the diseased site, the disease site has been identified.

### Supplementary Information

**Additional file 1: Figure S1.** Samples of different types of lesions in the used dataset. **Table S1.** The comparisons of numerous studies in WCE video classification using deep networks. **Table S2.** Proposed 2D-CNN characteristics. **Table S3.** Proposed 3D-CNN characteristics

## Data Availability

The datasets used and analyzed during the current study are available from the corresponding author upon reasonable request.

## Abbreviations

2D-CNN: Two-dimensional deep convolutional neural network
3D-CNN: Three-dimensional deep convolutional neural network
3DFCN: Three-dimensional fully convolutional network
CAD: Computer-aided diagnostic
GI: Gastrointestinal
GRU: Gated recurrent unit
LSTM: Long Short-Term Memory
NPV: Negative predictive value
PPV: Positive predictive value
ReLU: Rectified linear unit
RGB: Red green blue
SVM: Support vector machine
WCE: Wireless capsule endoscopy
