# Supplementary material for: Wireless capsule endoscopy multiclass classification using three-dimensional deep convolutional neural network model
Source: Biomed Eng Online. 2023 Dec 15;22:124. doi: 10.1186/s12938-023-01186-9 (PMC10722702; doi:10.1186/s12938-023-01186-9)
Supplement: Supplementary file 1 — Additional file 1: Figure S1. Samples of different types of lesions in the used dataset. Table S1. The comparisons of numerous studies in WCE video classification using deep networks. Table S2. Proposed 2D-CNN characteristics. Table S3. Proposed 3D-CNN characteristics [file 12938_2023_1186_MOESM1_ESM.docx]

**Additional file**

**Wireless capsule endoscopy multiclass classification using three-dimensional deep convolutional neural network model**

Mehrdokht Bordbar^1^, Mohammad Sadegh Helfroush^1^*, Habibollah Danyali^1^ and Fardad Ejtehadi^2^

*Correspondence: ms_helfroush@sutech.ac.ir;

^1^ Department of Electrical Engineering, Shiraz University of Technology, Shiraz, Iran

^2^ Department of Internal Medicine, Gastroenterohepatology Research Center, School of Medicine, Shiraz University of Medical Sciences, Shiraz, Iran

*BioMedical Engineering OnLine*

**Figure S1. Samples of different types of lesions in the used dataset**


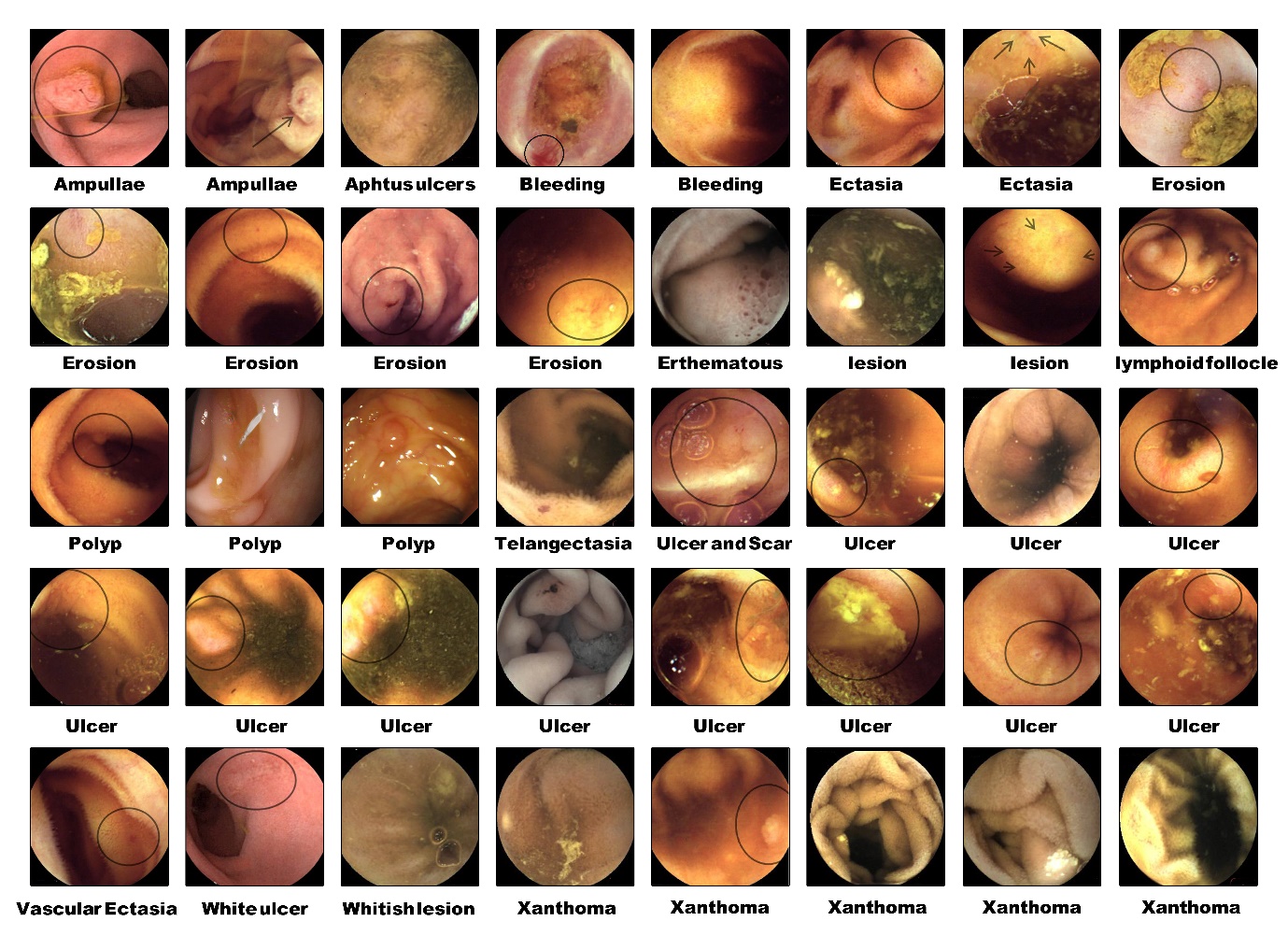


**Table S1. The comparisons of numerous studies in WCE video classification using deep networks**

| **Year** | **Method** | **Task** | **Dataset** | **Validation method** | **Results** |
| --- | --- | --- | --- | --- | --- |
| 2016 [[1](#_ENREF_1)] | 8 Layer CNN | Bleeding detection and classification | 10,000 frames  (Proprietary videos) | Cross validation, frame-based | F1 score: 99.5  Precision: 99.9  Recall: 99.2 |
| 2018 [[2](#_ENREF_2)] | AlexNet | Ulcers and erosion detection and classification | 21,160 frames  (Proprietary videos) | Cross validation, frame-based | Accuracy: 95.0  Sensitivity: 95.2  Specificity: 95.7 |
| 2019 [[3](#_ENREF_3)] | AlexNet, GooglNet | Ulcers detection and classification | 1,875 frames  (Proprietary videos) | Split dataset, frame-based | Accuracy: 100  Sensitivity: 100  Specificity: 100 |
| 2019 [[4](#_ENREF_4)] | Single Shot Multi-Box  Detector | Angioectasia detection and classification | 2,237 frames  (Proprietary videos) | Split dataset, frame-based | Accuracy: 98.7  Sensitivity: 98.8  Specificity: 98.4 |
| 2019 [[5](#_ENREF_5)] | Single Shot Multi-Box  Detector | Ulcers and erosion detection and classification | 1,580 frames  (Proprietary videos) | Split dataset, disease-based | Accuracy: 90.8  Sensitivity: 88.2  Specificity: 90.9 |
| 2020 [[6](#_ENREF_6)] | Inception-ResNet-v2 | Significant frames classification | 210,100 frames  (Proprietary videos) | Cross validation, frame-based & disease-based | Accuracy: 98.3  Sensitivity: 96.0  Specificity: 99.5 |
| 2021 [[7](#_ENREF_7)] | AlexNet + Triplet Network | Abnormality detection and classification | 5,306 frames  (Proprietary videos) | Split dataset, frame-based | Accuracy: 90.8  Sensitivity: 91.4  Specificity: 90.9 |
| 2021 [[8](#_ENREF_8)] | MobileNet + CNN | Bleeding detection and classification | 1650 frames  (Proprietary videos) | Cross validation, frame-based | Accuracy: 99.3  Sensitivity: 99.4  Precision: 100 |
| 2021 [[9](#_ENREF_9)] | Inception-Resnet-V2 | Multiple disease detection and classification | 400,000 frames  (Proprietary videos) | Split dataset, frame-based | Accuracy: 97.9  Sensitivity: 97.2  Specificity: 98.7 |
| 2021 [[10](#_ENREF_10)] | Backbone networks + SVM (VGG19, InceptionV3, ResNet50) | Bleeding detection and classification | KID Dataset 2 [[11](#_ENREF_11)] , MICAAI 2017 [[12](#_ENREF_12)] | Cross validation, frame-based | Accuracy: 98.2, 95.7  Sensitivity: 98.7, 95.8  Specificity: 97.3, 95.5 |
| 2022 [[13](#_ENREF_13)] | Backbone networks + Dense layers (Efficientnet, DenseNet, Xception) | Multiple disease detection and classification | Kvasir [[14](#_ENREF_14)] | Split dataset, frame-based | Accuracy: 94.8 |

**Table S2. Proposed 2D-CNN characteristics**

| **Layers arrange and their specifications** | | |
| --- | --- | --- |
| 1 | Image input | 224×224×3 images with “zerocenter” normalization |
| 2 | Convolution | 32 3×3×3 convolutions with stride [1 1] and padding “same” |
| 3 | Batch normalization | Batch normalization with 32 channels |
| 4 | ReLU | ReLU activation function |
| 5 | Max pooling | 2×2 max pooling with stride [2 2] and “zero” padding |
| 6 | Dropout | Dropout with 0.4 rate |
| 7 | Convolution | 64 3×3×32 convolutions with stride [1 1] and padding “same” |
| 8 | Batch normalization | Batch normalization with 64 channels |
| 9 | ReLU | ReLU activation function |
| 10 | Max pooling | 2×2 max pooling with stride [2 2] and “zero” padding |
| 11 | Dropout | Dropout with 0.4 rate |
| 12 | Convolution | 128 3×3×64 convolutions with stride [1 1] and padding “same” |
| 13 | Batch normalization | Batch normalization with 128 channels |
| 14 | ReLU | ReLU activation function |
| 15 | Max pooling | 2×2 max pooling with stride [2 2] and “zero” padding |
| 16 | Dropout | Dropout with 0.4 rate |
| 17 | Convolution | 256 3×3×128 convolutions with stride [1 1] and padding “same” |
| 18 | Batch normalization | Batch normalization with 256 channels |
| 19 | ReLU | ReLU activation function |
| 20 | Max pooling | 2×2 max pooling with stride [2 2] and “zero” padding |
| 21 | Dropout | Dropout with 0.4 rate |
| 22 | Convolution | 512 3×3×256 convolutions with stride [1 1] and padding “same” |
| 23 | Batch normalization | Batch normalization with 512 channels |
| 24 | ReLU | ReLU activation function |
| 25 | Max pooling | 2×2 max pooling with stride [2 2] and “zero” padding |
| 26 | Dropout | Dropout with 0.4 rate |
| 27 | Fully connected | 1 fully connected layer after the flatten filter |
| 28 | Dropout | Dropout with 0.4 rate |
| 29 | Softmax | Softmax layer with 3 output classes |
| **Training options** | | |
|  | Max epochs | 150 |
|  | Batch size | 32 |
|  | Learning rate | 0.0001 |
|  | Decay rate | 0.0001 |
|  | Solver for training network | Adam optimizer |
|  | Loss function | Cross-entropy |
|  | Total trainable parameters | 7,992,131 |

**Table S3. Proposed 3D-CNN characteristics**

| **Layers arrange and their specifications** | | |
| --- | --- | --- |
| 1 | Image input | 224×224×3×16 images with “zerocenter” normalization |
| 2 | Convolution | 32 3×3×3×3 convolutions with stride [1 1 1] and padding “same” |
| 3 | Batch normalization | Batch normalization with 32 channels |
| 4 | ReLU | ReLU activation function |
| 5 | Max pooling | 2×2×1 max pooling with stride [2 2 2] and “zero” padding |
| 6 | Dropout | Dropout with 0.4 rate |
| 7 | Convolution | 64 3×3×3×32 convolutions with stride [1 1 1] and padding “same” |
| 8 | Batch normalization | Batch normalization with 64 channels |
| 9 | ReLU | ReLU activation function |
| 10 | Max pooling | 2×2×2 max pooling with stride [2 2 2] and “zero” padding |
| 11 | Dropout | Dropout with 0.4 rate |
| 12 | Convolution | 128 3×3×3×64 convolutions with stride [1 1 1] and padding “same” |
| 13 | Batch normalization | Batch normalization with 128 channels |
| 14 | ReLU | ReLU activation function |
| 15 | Max pooling | 2×2×2 max pooling with stride [2 2 2] and “zero” padding |
| 16 | Dropout | Dropout with 0.4 rate |
| 17 | Convolution | 256 3×3×3×128 convolutions with stride [1 1 1] and padding “same” |
| 18 | Batch normalization | Batch normalization with 256 channels |
| 19 | ReLU | ReLU activation function |
| 20 | Max pooling | 2×2×2 max pooling with stride [2 2 2] and “zero” padding |
| 21 | Dropout | Dropout with 0.4 rate |
| 22 | Convolution | 512 3×3×3×256 convolutions with stride [1 1 1] and padding “same” |
| 23 | Batch normalization | Batch normalization with 512 channels |
| 24 | ReLU | ReLU activation function |
| 25 | Max pooling | 2×2×2 max pooling with stride [2 2 2] and “zero” padding |
| 26 | Dropout | Dropout with 0.4 rate |
| 27 | Fully connected | 1 fully connected layer after the flatten filter |
| 28 | Dropout | Dropout with 0.4 rate |
| 29 | Softmax | Softmax layer with 3 output classes |
| **Training options** | | |
|  | Max epochs | 150 |
|  | Batch size | 32 |
|  | Learning rate | 0.0001 |
|  | Decay rate | 0.0001 |
|  | Solver for training network | Adam optimizer |
|  | Loss function | Cross-entropy |
|  | Total trainable Parameters | 49,722,593 |

**References:**

1. Jia, X. and M.Q.-H. Meng. *A deep convolutional neural network for bleeding detection in wireless capsule endoscopy images*. in *2016 38th Annual International Conference of the IEEE Engineering in Medicine and Biology Society (EMBC)*. 2016. IEEE.

2. Fan, S., et al., *Computer-aided detection of small intestinal ulcer and erosion in wireless capsule endoscopy images.* Physics in Medicine & Biology, 2018. **63**(16): p. 165001.

3. Alaskar, H., et al., *Application of convolutional neural networks for automated ulcer detection in wireless capsule endoscopy images.* Sensors, 2019. **19**(6): p. 1265.

4. Tsuboi, A. and S. Oka, *Artificial intelligence using a convolutional neural network for automatic detection of small-bowel angioectasia in capsule endoscopy images.* 2020. **32**(3): p. 382-390.

5. Aoki, T., et al., *Automatic detection of erosions and ulcerations in wireless capsule endoscopy images based on a deep convolutional neural network.* Gastrointestinal endoscopy, 2019. **89**(2): p. 357-363. e2.

6. Park, J., et al., *Artificial intelligence that determines the clinical significance of capsule endoscopy images can increase the efficiency of reading.* PLoS One, 2020. **15**(10): p. e0241474.

7. Adewole, S., et al. *Lesion2Vec: Deep Meta Learning for Few-Shot Lesion Recognition in Capsule Endoscopy Video*. in *Proceedings of the Future Technologies Conference (FTC) 2021, Volume 2*. 2022. Cham: Springer International Publishing.

8. Rustam, F., et al., *Wireless Capsule Endoscopy Bleeding Images Classification Using CNN Based Model.* IEEE Access, 2021. **9**: p. 33675-33688.

9. Kim, S.H., et al., *Efficacy of a comprehensive binary classification model using a deep convolutional neural network for wireless capsule endoscopy.* Scientific Reports, 2021. **11**(1): p. 17479.

10. Caroppo, A., A. Leone, and P. Siciliano, *Deep transfer learning approaches for bleeding detection in endoscopy images.* Computerized Medical Imaging and Graphics, 2021. **88**: p. 101852.

11. Koulaouzidis, A., et al., *KID Project: an internet-based digital video atlas of capsule endoscopy for research purposes.* Endoscopy international open, 2017. **5**(06): p. E477-E483.

12. Coelho, P., et al. *A Deep Learning Approach for Red Lesions Detection in Video Capsule Endoscopies*. in *Image Analysis and Recognition*. 2018. Cham: Springer International Publishing.

13. Su, Q., et al., *Deep convolutional neural networks with ensemble learning and transfer learning for automated detection of gastrointestinal diseases.* Computers in Biology and Medicine, 2022. **150**: p. 106054.

14. Pogorelov, K., et al. *Kvasir: A multi-class image dataset for computer aided gastrointestinal disease detection*. in *Proceedings of the 8th ACM on Multimedia Systems Conference*. 2017.
